# Imbalance of the Nerve Growth Factor and Its Precursor as a Potential Biomarker for Diabetic Retinopathy

**DOI:** 10.1155/2015/571456

**Published:** 2015-03-17

**Authors:** B. A. Mysona, S. Matragoon, M. Stephens, I. N. Mohamed, A. Farooq, M. L. Bartasis, A. Y. Fouda, A. Y. Shanab, D. G. Espinosa-Heidmann, A. B. El-Remessy

**Affiliations:** ^1^Program in Clinical and Experimental Therapeutics, College of Pharmacy, University of Georgia, Augusta, GA 30912, USA; ^2^Culver Vision Discovery Institute, Georgia Regents University, Augusta, GA 30912, USA; ^3^Charlie Norwood Veterans Affairs Medical Center, Augusta, GA 30904, Augusta, USA

## Abstract

Our previous studies have demonstrated that diabetes-induced oxidative stress alters homeostasis of retinal nerve growth factor (NGF) resulting in accumulation of its precursor, proNGF, at the expense of NGF which plays a critical role in preserving neuronal and retinal function. This imbalance coincided with retinal damage in experimental diabetes. Here we test the hypothesis that alteration of proNGF and NGF levels observed in retina and vitreous will be mirrored in serum of diabetic patients. Blood and vitreous samples were collected from patients (diabetic and nondiabetic) undergoing vitrectomy at Georgia Regents University under approved IRB. Levels of proNGF, NGF, and p75^NTR^ shedding were detected using Western blot analysis. MMP-7 activity was also assayed. Diabetes-induced proNGF expression and impaired NGF expression were observed in vitreous and serum. Vitreous and sera from diabetic patients (*n* = 11) showed significant 40.8-fold and 3.6-fold increases, respectively, compared to nondiabetics (*n* = 9). In contrast, vitreous and sera from diabetic patients showed significant 44% and 64% reductions in NGF levels, respectively, compared to nondiabetics. ProNGF to NGF ratios showed significant correlation between vitreous and serum. Further characterization of diabetes-induced imbalance in the proNGF to NGF ratio will facilitate its utility as an early biomarker for diabetic complications.

## 1. Introduction

Diabetic retinopathy (DR), a leading cause of blindness in working age adults, is estimated to affect 101 million people worldwide [1]. Although DR is initially asymptomatic, chronic hyperglycemia caused by insufficient insulin damages the retinal microvasculature. Early insults include pericyte loss, microaneurysms, and leukostasis [2–4]. Dysfunction of the blood retinal barrier is evidenced by increased microvascular permeability and diabetic macular edema (DME), which lead to decreased visual acuity [5, 6]. Endothelial cell death and acellular capillary formation further impair the retinal blood supply leading to proliferative diabetic retinopathy (PDR) which is characterized by growth of fragile, leaky blood vessels and loss of vision [7, 8]. Current treatments for DME and PDR such as laser photocoagulation and anti-VEGF injections are invasive with considerable side effects [9–11]. Even though the overall risk of developing DR increases with duration of diabetes, poor glycemic control, and high blood pressure, the rate of development and severity of DR varies greatly from patient to patient [1, 12–14]. Several studies have identified morphological biomarkers associated with the progression of DR including changes in the multifocal electroretinogram, microaneurysm turnover, and subclinical edema [6, 15–22]. In addition, altered expression of cytokines, chemokines, and angiogenic and apoptotic related factors has been identified in the vitreous of patients with DME and PDR [23–27]. Despite these advances, we still do not have a reliable biomarker that is readily detectable in serum and that predicts the likelihood of a patient developing sight threatening complications of DR.

Changes in level of nerve growth factor (NGF) have been previously assessed in diabetic patients in relation to diabetic retinopathy and neuropathy [28–30]. NGF is traditionally released as the proform, proNGF, which is cleaved intracellularly by furins and extracellularly by several proteases including MMP-7 [31, 32]. While NGF binds to the tyrosine kinase receptor A (TrkA) to signal through prosurvival pathways, proNGF binds preferentially to p75^NTR^, which in combination with its coreceptor sortilin generally activates inflammatory and apoptotic pathways (reviewed in [33–35]). Our group has discovered that diabetes causes an imbalance of increased proNGF at the expense of mature NGF due to impaired MMP-7 activity in clinical and experimental diabetes [31]. Specifically, proNGF levels are elevated and NGF levels reduced in the aqueous humor of patients with PDR and in the vitreous of diabetic patients. In experimental STZ-diabetes rat model, decreases in NGF were associated with early retinal neurodegeneration [36–38]. Treatments that enhanced the levels of NGF either endogenously [31, 36] or by exogenous supplement of recombinant NGF protein [37, 39] prevented retinal neurodegeneration in models of diabetes. In the diabetic retina, increased expression of p75^NTR^, the preferred receptor of proNGF, exacerbates the effects of the proNGF/NGF imbalance [36, 38, 40] by favoring the activation of the proNGF/p75^NTR^ signaling pathways that are associated with increases in inflammatory mediators and vascular permeability [36, 38, 40].

In the present work, we performed a small pilot study investigating the feasibility of whether changes in proNGF/NGF levels observed in vitreous will be mirrored in serum and thus provide rationale to examine proNGF as a biomarker for diabetic retinopathy. This study includes an analysis of vitreous and serum samples from patients with PDR and from nondiabetic patients, all undergoing vitrectomy.

## 2. Subjects and Methods

### 2.1. Study Participants

Participants were recruited from the Georgia Regents Eye Care Center, Augusta, GA. All participants gave written informed consent and the study was governed under guidelines of the local institutional review board. A total of 9 nondiabetic and 11 diabetic participants were included in this study. Blood was drawn shortly before surgery. Vitreous was collected from participants undergoing pars plana vitrectomy. Characteristics of the study participants are summarized in Table 1.

### 2.2. Serum Sample Preparation

Whole blood was allowed to clot 60–90 minutes at room temperature and then was centrifuged at 3200 g for 15 minutes to separate serum from red blood cells. Serum blood glucose was tested using the ReliOn Ultima blood glucose meter (Abbott Diabetes Care, Inc., Alameda, CA). Serum aliquots were placed into cryovials and stored at −80°C for further analysis.

### 2.3. Vitreous Sample Preparation

Vitreous wash was collected, filtered, and concentrated. Vitreous was prefiltered through a combination Whatman 1 filter paper circle (GE Healthcare Biosciences, Pittsburgh, PA) fit to a Nalgene Rapid-Flow sterile filter unit (Thermo Fisher Scientific, Waltham, MA). Proteins 10 kilodaltons (kD) and greater were concentrated from the filtrate with a Corning Spin-X UF concentrator (Corning Inc., Corning, NY) and Amicon Ultra centrifugal filters (Merck Millipore, Darmstadt, Germany). Concentrated aliquots were placed into cryovials and stored at −80°C for further analysis.

### 2.4. Protein Estimation

Protein concentration of serum and vitreous was measured using Bio-Rad DC protein assay (Bio-Rad Laboratories, Inc., Hercules, CA).

### 2.5. Western Blot Sample Preparation

Serum and vitreous samples were diluted to a concentration of 3 *μ*g/*μ*L in RIPA buffer with protease inhibitor cocktail (Merck Millipore, Darmstadt, Germany), 1 mM phenylmethylsulfonyl fluoride (Sigma-Aldrich, St. Louis, MO), and halt phosphatase inhibitor cocktail (Thermo Scientific, Waltham, MA). Dilution of serum was critical so that samples could be boiled without coagulation. For Western blot, 35–50 *μ*g aliquots with Laemmli sample buffer (Bio-Rad Laboratories, Inc., Hercules, CA) plus beta mercaptoethanol (Sigma-Aldrich, St. Louis, MO) were boiled 10 minutes and then loaded on 15% polyacrylamide gels, which results in good separation of proteins in the 10 to 30 kD range. Thus, the larger abundant proteins such as albumin (66 kD) are held back in the upper portion of the gel away from the proteins that we are interested in (proNGF 26–32 kD, NGF 13 kD).

### 2.6. Western Blot Analysis

Samples of vitreous and serum were separated by SDS-PAGE, transferred to nitrocellulose membrane and stained with Ponceau S (Sigma-Aldrich, St. Louis, MO). After blocking for 15–30 minutes, membranes were probed overnight with primary antibodies per manufacturer's recommendation in 5% milk or 2% BSA followed by secondary antibody (1 : 5000 in 5% milk). Blots were developed using Pierce ECL Western Blot Substrate (Thermo Fisher Scientific, Rockford, IL) or Immoblion Western chemiluminescent HRP substrate (Merck Millipore, Darmstadt, Germany). Films were scanned and the band intensity was quantified using densitometry software (alphEaseFC) and then expressed as relative optical density (ROD) normalized to Ponceau S (due to lack of a specific loading control for serum and vitreous) and expressed as fold change from nondiabetic controls. Representative images chosen for Western blot were non-adjacent bands from the same blot.

### 2.7. Antibodies

The following antibodies were used for immunoblotting: rabbit polyclonal anti-NGF and anti-proNGF (Alomone Labs, Israel) and rabbit polyclonal p75^NTR^ (1 : 5000), a kind gift from Dr. Bruce Carter, Vanderbilt University, Nashville, TN. Secondary antibodies were horseradish peroxidase-conjugated goat anti-rabbit (Calbiochem, La Jolla, CA).

### 2.8. MMP-7 Activity

The activity of matrix metalloproteinase 7 (MMP-7) was determined using the Sensolyte 490 generic MMP-7 fluorimetric assay kit (AnaSpec, EGT Corporation, Fremont, CA) per the manufacturer's instructions and as described by our group previously [31].

### 2.9. Data Analysis

Graphs were prepared with the aid of GraphPad Prism 6.04 (GraphPad Software Inc., La Jolla, CA). Results were expressed as mean ± SEM. Normality of data was verified by the D'Agostino and Pearson omnibus normality test. Normal data sets were compared using unpaired* t*-test with or without Welch's correction for unequal variances. Nonparametric data sets were evaluated using the Mann-Whitney test. After eliminating outliers, correlation between normalized proNGF/NGF expression ratio in diabetic vitreous and serum was calculated using the Pearson coefficient followed by Deming linear regression between groups with different standard deviations. Outliers were determined to be all proNGF/NGF ratios outside the range of the median ±1.5 × (interquartile range). Data analysis was performed using GraphPad Prism 6.04 and JMP Pro 10.0.1 Release 2 software (SAS, Cary, NC). Significance was defined as *P* < 0.05.

## 3. Results

### 3.1. Study Participants

A total of 20 participants, both males and females of various ethnic backgrounds, were recruited from the Georgia Regents Eye Care Center, Augusta, GA. The 9 nondiabetic control patients had an average age of 55.9 ± 5.6 years and were undergoing pars plana vitrectomy for retinal detachment or macular pucker. The 11 diabetic participants were undergoing pars plana vitrectomy for retinal detachment as a complication of PDR and were primarily type 2 diabetic with an average age of 48.5 ± 4.2 years. HbA1c values were available for half of the diabetic participants and averaged 8.3% ± 0.8. Characteristics of the participants appear in Table 1.

### 3.2. Blood Glucose Levels Are Higher in Diabetic Participants

**Figure 1:**
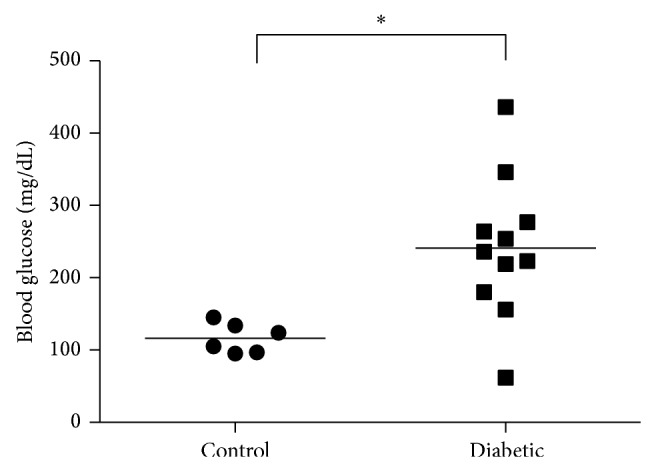
Blood glucose higher in diabetic patients. On average, diabetic participants had significantly higher glucose concentration of 241 mg/dL ± 30 than nondiabetic control participants 117 mg/dL ± 9 in their serum (*N* = 6–11, ^*^ *P* < 0.05).

Screening of participant blood glucose levels showed that all but one of the diabetic patients had significantly higher serum glucose levels than their nondiabetic counterparts (Figure 1). On average, blood glucose in serum from diabetic participants was 241 mg/dL ± 30 while nondiabetic control participants had serum glucose values of 117 mg/dL ± 9.

### 3.3. ProNGF Increases Are Consistent in Vitreous and Serum

**Figure 2:**
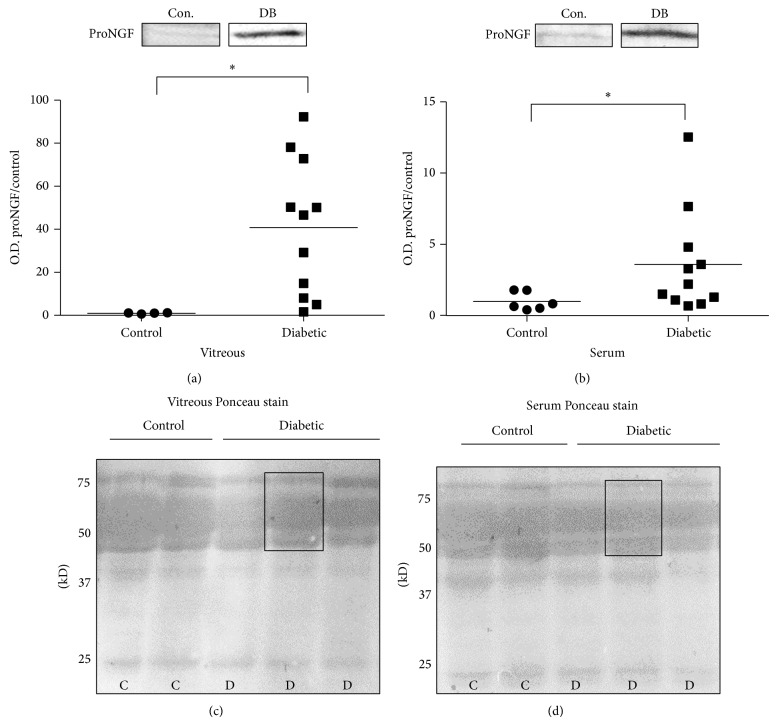
ProNGF increases are consistent in vitreous and serum. Representative blots and statistical analysis of proNGF protein expression are shown. ProNGF band intensities in vitreous and serum of diabetic (DB) and nondiabetic control (Con.) participants were normalized to Ponceau S and respective control group. (a) ProNGF expression is significantly elevated in vitreous of diabetic, 40.8-fold ± 9.5, relative to nondiabetic control group (*N* = 4–11, ^*^ *P* < 0.05). (b) In serum, proNGF expression is significantly increased, 3.6-fold ±1.1, compared to nondiabetic controls (*N* = 6–11, ^*^ *P* < 0.05). Typical image of Ponceau staining for (c) vitreous blots and (d) serum blots showing the equal loading of control and diabetic samples as well as the area selected for intensity measurements.

**Figure 3:**
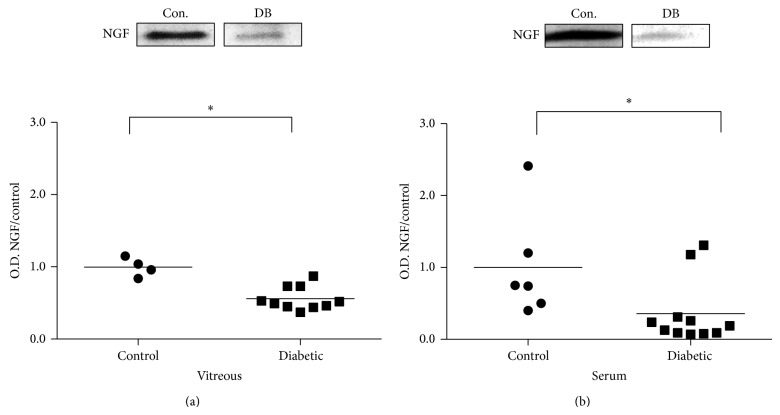
NGF decreases are consistent in vitreous and serum. Representative blots and statistical analysis of NGF protein expression are shown. NGF band intensities in vitreous and serum of diabetic (DB) and nondiabetic control (Con.) participants were normalized to Ponceau S and respective control group. (a) NGF expression is significantly reduced in diabetic vitreous, 0.56 ± 0.05, compared to nondiabetic control group, 1.00 ± 0.07 (*N* = 4–10, ^*^ *P* < 0.05). (b) A significant decrease in NGF expression occurs in diabetic serum, 0.36 ± 0.13, compared to nondiabetic controls 1.00 ± 0.30 (*N* = 6–11, ^*^ *P* < 0.05).

Statistical analysis of Western blot band intensity normalized to Ponceau S staining showed that the observed increases in proNGF in vitreous occurred also in serum, albeit, to a lesser extent. In diabetic vitreous, proNGF was significantly elevated 40.8-fold ±9.5. In serum, the trend towards increased proNGF expression was not as dramatic but still significant with a 3.6-fold ±1.1 elevation in proNGF expression compared to nondiabetic controls (Figures 2(a) and 2(b)). A typical example of the Ponceau S signal demonstrates that this staining can be used as a crude measure of evaluating the equal loading between control and diabetic samples from vitreous and serum (Figures 3(a) and 3(b)). The Ponceau S signal was determined by measuring band intensity of a rectangle width of the band from 50 to 75 kD (Figures 2(c) and 2(d)).

### 3.4. NGF Decreases Are Consistent in Vitreous and Serum

Statistical analysis of Western blot band intensity normalized to Ponceau S staining showed that the proNGF/NGF imbalance in vitreous occurred also in serum, albeit, to a lesser extent. In diabetic vitreous, NGF expression was significantly decreased 0.56 ± 0.05 compared to the nondiabetic control group (Figure 3(a)). NGF expression was significantly decreased in serum of diabetic participants, 0.36 ± 0.13, relative to nondiabetic controls (Figure 3(b)).

### 3.5. Imbalance of ProNGF/NGF Ratio Is Consistent in Vitreous and Serum

**Figure 4:**
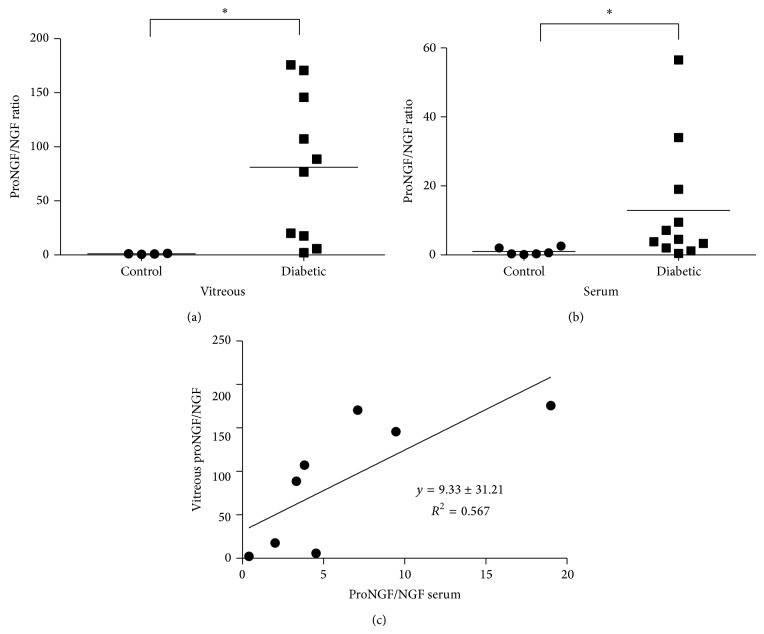
ProNGF/NGF ratio in vitreous correlated to proNGF/NGF ratio in serum. (a) In vitreous, the expression ratio of proNGF to NGF in individual diabetic participants was significantly higher than nondiabetic control participants (*N* = 4–10, ^*^ *P* < 0.05). (b) In serum expression ratio of proNGF to NGF in individual diabetic participants was also significantly higher than nondiabetic control participants (*N* = 6–11, ^*^ *P* < 0.05). (c) Deming linear regression of proNGF/NGF ratios in vitreous as a function of the ratio in serum of diabetic participants had a slope of 9.33 ± 3.33 and an intercept of 31.21 ± 27.65. The linear correlation was significant with a Pearson coefficient of *R* ^2^ = 0.567 (*N* = 8, *P* < 0.05).

Next, we evaluated the usefulness of the proNGF/NGF expression ratio as a measure of the proNGF/NGF imbalance in vitreous and serum. In vitreous, the proNGF to NGF expression ratio in individual diabetic participants was significantly 81.0-fold higher than in nondiabetic control participants (Figure 4(a)). In serum the proNGF/NGF expression ratio was significantly increased by a more modest 12.9-fold compared to serum of nondiabetic control participants (Figure 4(b)). After elimination of two diabetic participants because of very high proNGF/NGF ratios determined to be outliers, correlation and linear regression were performed on the remaining proNGF/NGF ratios. Deming linear regression of proNGF/NGF ratios in vitreous as a function of the ratio in serum of diabetic participants resulted in a linear correlation described by the equation *y* = 9.33*x* + 31.21 with a Pearson coefficient of *R* ^2^ = 0.567 (Figure 4(c)).

### 3.6. MMP-7 Activity Decreased in Vitreous but Not in Serum

**Figure 5:**
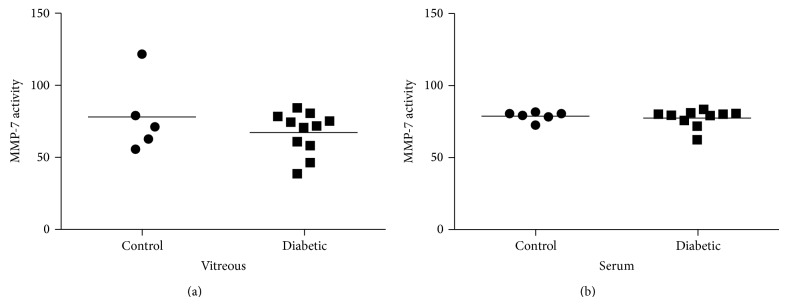
MMP-7 activity is decreased in vitreous but not in serum. (a) In vitreous, MMP-7 activity was decreased in diabetic (66.6 ± 4.4) compared to nondiabetic control group (77.5 ± 11.7). (b) In serum, MMP-7 activity detected in the diabetic groups (77.3 ± 2.0) was similar to the activity in nondiabetic controls (78.7 ± 1.3).

The enzyme MMP-7 is known to cleave proNGF to form the mature neurotrophin NGF [32]. MMP-7 proteolytic activity, measured in relative fluorescent units (RFU), was reduced in diabetic vitreous to 66.6 RFU ± 4.4, compared to the nondiabetic control group, 77.5 RFU ± 11.7, but the difference between groups was not significant (Figure 5(a)). Interestingly, in serum, MMP-7 activity detected in the diabetic group, 77.3 RFU ± 2.0, was similar to the activity in nondiabetic controls, 78.7 RFU ± 1.3 (Figure 5(b)).

### 3.7. Shedding of p75^NTR^ Receptor Is Consistent in Vitreous and Serum

**Figure 6:**
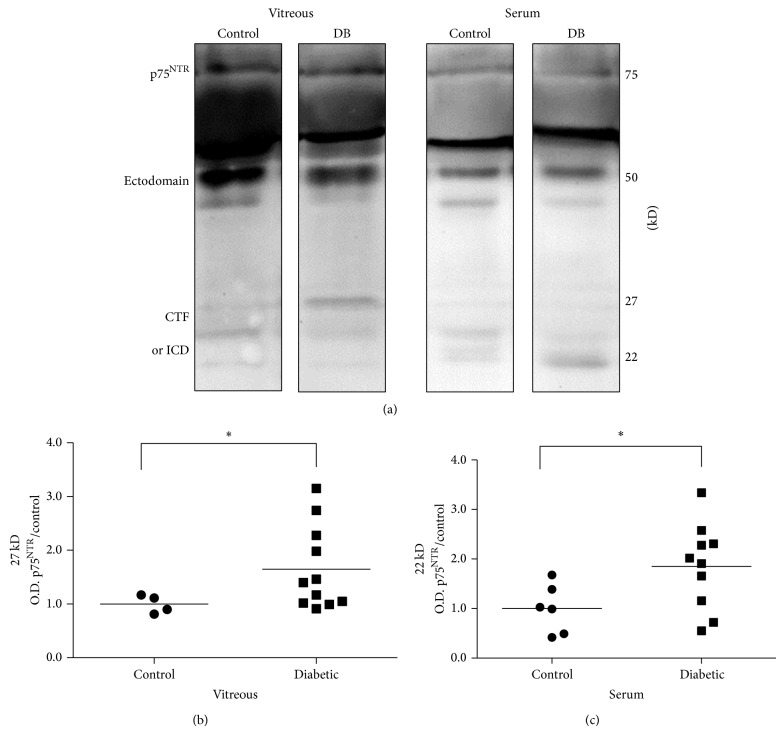
Shedding of p75^NTR^ is consistent in vitreous and serum. (a) Representative bands show p75^NTR^ expression in vitreous and serum for diabetic compared to nondiabetic control groups. The full length p75^NTR^ (75 kD) and receptor ectodomain (50 kD) had similar levels of expression in control and diabetic (DB) groups of both vitreous and serum. The possible proteolytic C terminal fragment (CTF) and intracellular domain (ICD) appeared at 27 kD and 22 kD. Differences in expression patterns between vitreous and serum as well as between diabetic and control groups were evident for both CTF and ICD. (b) In vitreous, 27 kD p75^NTR^ receptor fragment was significantly increased in diabetic (1.65-fold ± 0.23) compared to nondiabetic control group (*N* = 4–11, ^*^ *P* < 0.05). (c) In serum, a significant increase in 22 kD p75^NTR^ occurred in diabetic samples (1.85-fold ± 0.30) compared to nondiabetic controls (*N* = 6–10, ^*^ *P* < 0.05).

Expression of p75^NTR^ receptor in vitreous and serum samples from diabetic and control participants was analyzed by Western blot. Signaling via p75^NTR^ involves a sequence of proteolytic events including ectodomain shedding and regulated intramembrane proteolysis [41–43]. Accordingly, multiple sized p75^NTR^ variants were detected by WB. Representative blots (Figure 6(a)) of p75^NTR^ showed three main size variants including full length p75^NTR^ (75 kD), the receptor ectodomain (50 kD), a 27 kD possible C-terminal fragment (CTF), and a 22 kD possible intracellular domain (ICD) fragment. At the present time, distinguishing the exact identity of these smaller fragments is not possible. Statistical analysis of band intensities showed that, in vitreous, 27 kD p75^NTR^ possible CTF fragment was significantly increased in diabetic (1.65-fold ± 0.23) compared to nondiabetic control group (Figure 6(b)). In serum, a significant increase in 22 kD p75^NTR^ possible ICD fragment occurred in diabetic (1.85 fold ± 0.30) compared to nondiabetic controls (Figure 6(c)).

**Figure 7:**
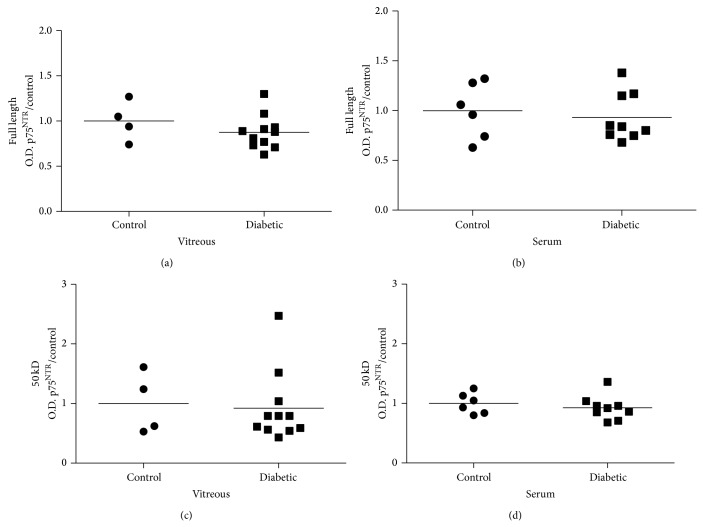
Expression of p75^NTR^ receptor is consistent in vitreous and serum. Results are shown for p75^NTR^ expression in vitreous and serum of diabetic (DB) and control participants normalized to Ponceau S and respective controls. Full length p75^NTR^ receptor (75 kD) was not significantly different in diabetic sample compared to control groups in either (a) vitreous (*N* = 4–11) or (b) serum (*N* = 6–9). The p75^NTR^ ectodomain (50 kD) was also not significantly different in diabetic compared to control groups in (c) vitreous (*N* = 4–11) or (d) serum (*N* = 6–9).

Statistical analysis of band intensities showed that full length p75^NTR^ receptor (75 kD) was not significantly different in diabetic compared to control groups in either vitreous or serum (Figures 7(a) and 7(b)). The p75^NTR^ ectodomain (50 kD) was also not significantly different in diabetic compared to control groups in vitreous or serum (Figures 7(c) and 7(d)).

## 4. Discussion

The results of this pilot study shed light on the relationship between the diabetes-induced proNGF/NGF imbalance in vitreous and serum. Three main findings arose from this work. First, on average, the increased proNGF and decreased NGF expression in diabetic vitreous was also observed in diabetic serum. There was significant and positive correlation of the ratio of proNGF/NGF in vitreous to serum in PDR patients. Second, MMP-7 activity in vitreous of participants with PDR was slightly lower than in nondiabetic control subjects; however, no difference between groups was observed in serum. Third, proteolytic shedding of p75^NTR^ receptor fragments was increased in both diabetic vitreous and serum compared to nondiabetics. Together, these results support the feasibility and rationale of investigating the increased serum proNGF/NGF ratio as a biomarker of DR.

While there has been interest in correlating changes in NGF levels with diabetic complications, previous studies have used the enzyme-linked immunosorbent assay (ELISA) that cannot differentiate between NGF and its precursor form, proNGF. These studies showed that diabetes increases serum NGF levels and omitted the impact of diabetes on the homeostasis between proNGF and NGF [28–30]. In the present study, this problem was circumvented by separating serum and vitreous samples by Western blot and by utilizing an anti-proNGF antibody that specifically recognizes the “pro” region of proNGF which can exist as 26, 32, or 40 kD forms depending on the glycosylation state [44–46]. To avoid any confusion between the NGF dimer and 26 kD proNGF, the smaller NGF monomer (13 kD) was evaluated in our studies. The results of our pilot study showed that serum from diabetic participants with PDR had a significant 3.6-fold increase in proNGF expression compared to a more dramatic increase of 40.8-fold in the diabetic vitreous relative to nondiabetic control group. In addition to elevated serum proNGF, low levels of serum NGF in diabetic participants exacerbated the effects of increased proNGF. NGF levels in participants with PDR were decreased significantly to 0.36 ± 0.13 of control levels in serum and 0.56 ± 0.05 of control levels in vitreous compared to the nondiabetic control group. The present study resulted in the novel finding that a vitreous proNGF/NGF ratio is positively correlated with its ratio in serum of PDR patients (Figure 4). Increases in proNGF and decreases in NGF in vitreous from PDR patients lend further support to our previous findings in ocular fluids of diabetic patients [31] and experimental diabetes [36, 38, 40]. One of the caveats in studying vitreous protein changes in PDR is that protein levels will be higher in the diabetic vitreous due to leakage of blood into the vitreous [27]. This effect is partially corrected by the loading of equal amounts of protein for each sample; however, the possibility remains that some of the vitreal protein expression changes may be influenced by leakage of blood into the retina. Overall, the large increase in proNGF expression in vitreous, decrease in NGF expression, and different banding patterns for p75^NTR^ fragment expression in vitreous versus serum lead us to believe that these trends are linked to protein expression changes in the vitreous. Although proNGF is better known for its roles in neuronal development, CNS injury, and neurodegenerative diseases, proNGF and NGF are increasingly recognized for their important signaling functions in such diverse organs as retina, brain, pancreas, kidney, thyroid, testes, immune system, and vasculature [30, 31, 46–56]. Thus, diabetes-induced alterations in the proNGF/NGF imbalance in serum may well reflect combined contributions from the retina, other organs, the immune system, and the vasculature.

The second finding of this pilot study was that although MMP-7 activity was lower in vitreous of participants with PDR, no apparent activity difference between groups was observed in serum. Although not statistically significant, lower MMP-7 activity in vitreous agrees with previous findings that MMP-7 activity is significantly reduced in aqueous humor of patients with PDR and that MMP-7 protein expression is decreased in diabetic rat retinal lysates [31]. Similar results are reported in kidney with MMP-7 activity and protein expression reduced under conditions of diabetic nephropathy [57]. The correlation, however, between tissue levels of MMP-7 versus the systemic environment of serum is not always straightforward. Two separate studies have documented that serum levels of MMP-7 are higher in diabetic subjects [58, 59]. The dichotomy between MMP-7 action in diabetic serum and retina is further illustrated by findings that the cholesterol lowering drug atorvastatin reduces MMP-7 levels in diabetic serum but increases its expression in diabetic rat retina [31, 59]. Future studies on mechanisms controlling MMP-7 expression in different organ systems would be valuable.

The third finding of this study was that altered expression of p75^NTR^ proteolytic fragments occurred in diabetic vitreous and serum. Since p75^NTR^, the preferred receptor for proNGF, lacks intrinsic kinase activity, the p75^NTR^ receptor signals via ectodomain shedding and regulated intramembrane proteolysis [41–43]. This results in detecting full length p75^NTR^ (75 kD), the receptor ectodomain (50 kD), and smaller fragments, such as the C-terminal fragment (CTF, 27 kD) and p75 intracellular domain (ICD, 22 kD). Western blot analysis of vitreous and serum did not show any significant differences in full length p75^NTR^ or the ectodomain expression between diabetic and control groups (Figure 7). Although expression of full length p75^NTR^ receptor has been reported to be elevated in the plasma of type 2 diabetic rats, our results correspond more closely to a recent study in diabetic patients showing only minor changes in p75^NTR^ expression [60, 61]. We believe that the 50 kD band (Figures 6 and 7) is most likely the ectodomain fragment which should be found in extracellular mostly acellular environment of the vitreous or serum. Previous reports proposed that this fragment might also be a smaller unglycosylated form of p75^NTR^ [41–43]. Our most intriguing observation regarding p75^NTR^ expression was the apparent variations in CTF and ICD expression in vitreous and serum as well as between diabetic and control groups. The p75^NTR^ CTF and ICD fragments have most commonly been identified in cell culture studies and the reported sizes of these fragments vary somewhat depending on the cell type and experimental conditions used.

In summary, we hypothesized that the ratio of proNGF to NGF more correctly indicates homeostasis in the retina than the level of either neurotrophin alone. High proNGF expression may not necessarily be detrimental if it is accompanied by high levels of NGF. In fact, the linear correlation between either proNGF or NGF levels alone in vitreous with serum did not yield a significant correlation. Using the proNGF/NGF ratio, however, did result in a significant positive Pearson coefficient of *R* ^2^ = 0.567 showing a correlation between proNGF/NGF ratios in vitreous and serum. Deming linear regression showed that serum proNGF/NGF expression ratios can be used to predict vitreous proNGF/NGF ratios by the equation *y* = 9.33*x* + 31.21. The current study is limited by a small number of participants and that all the diabetic patients had PDR, the lattermost severe stage of this disease. A study focusing on the association of serum proNGF/NGF imbalance with early microvascular indicators of DR such as microaneurysm formation identified by fundus photography or macular edema visualized by OCT would be informative. The consistent imbalance of proNGF/NGF ratio in both ocular fluids and serum in diabetic patients suggests that treatments to restore NGF levels represent potential therapeutic strategies for diabetic complications. We and others have demonstrated protective effects of restoring NGF in experimental models of diabetes [31, 36, 37, 39]. Clinically, a recent report showed the safety of an exogenous human recombinant NGF in Phase I study, suggesting the feasibility of examining its efficacy as the next step [62]. The discovery of biomarkers to aid in the identification of patients most likely to develop severe DME and PDR is essential for better treatment of this disease. Initial data presented here suggest that on average the proNGF/NGF imbalance in serum was reflective of the proNGF/NGF imbalance in vitreous of patients with PDR. Further investigations to determine if the proNGF/NGF imbalance can be correlated with the progression of DR will be valuable to determine if the proNGF/NGF imbalance can truly be a biomarker of DR.

## Figures and Tables

**(a):** 

Patient	Type	Age	Sex	Ethnicity	Sample	Retinal pathology
1	Control	65	Female	White	Both	Macular hole
2	Control	72	Male	White	Both	Macular pucker
3	Control	18	Female	Black	Vitreous	Retinal detachment
4	Control	59	Male	White	Serum	Retinal detachment
5	Control	60	Male	White	Serum	Retinal detachment
6	Control	70	Male	Black	Vitreous	Retinal detachment, globe injury
7	Control	40	Male	White	Serum	Retinal detachment
8	Control	62	Female	Black	Vitreous	Macular hole
9	Control	57	Male	White	Serum	Retinal detachment

**(b):** 

Patient	Type	Age	Sex	Ethnicity	Sample	HbA1c	Retinal pathology
10	Diabetic	46	Female	Black	Both	Unknown	PDR
11	Diabetic	75	Male	White	Both	8.7	PDR
12	Diabetic	57	Female	Black	Both	9.7	PDR
13	Diabetic	38	Female	Black	Both	11.3	PDR
14	Diabetic	51	Female	Black	Both	6.8	PDR
15	Diabetic	58	Female	White	Both	Unknown	PDR
16	Diabetic	31	Female	Black	Both	Unknown	PDR
17	Diabetic	55	Male	Black	Both	Unknown	PDR
18	Diabetic	50	Female	White	Both	6.8	PDR
19	Diabetic	24	Female	Black	Both	6.4	PDR
20	Diabetic	49	Male	Black	Both	Unknown	PDR
